# Transport of Non-Spherical Particles in Square Microchannel Flows: A Review

**DOI:** 10.3390/mi12030277

**Published:** 2021-03-07

**Authors:** Tohme Tohme, Pascale Magaud, Lucien Baldas

**Affiliations:** Institut Clément Ader (ICA), INSA, ISAE-SUPAERO, Mines-Albi, UPS, Université de Toulouse, 3 rue Caroline Aigle, 31400 Toulouse, France; tohme@insa-toulouse.fr (T.T.); pascale.magaud@iut-tlse3.fr (P.M.)

**Keywords:** inertial migration, non-spherical particles, rotational behavior, particle transport, particle-laden microflows

## Abstract

Understanding the behavior of a single particle flowing in a microchannel is a necessary step in designing and optimizing efficient microfluidic devices for the separation, concentration, counting, detecting, sorting, or mixing of particles in suspension. Although the inertial migration of spherical particles has been deeply investigated in the last two decades, most of the targeted applications involve shaped particles whose behavior in microflows is still far from being completely understood. While traveling in a channel, a particle both rotates and translates: it translates in the streamwise direction driven by the fluid flow but also in the cross-section perpendicular to the streamwise direction due to inertial effects. In addition, particles’ rotation and translation motions are coupled. Most of the existing works investigating the transport of particles in microchannels decouple their rotational and lateral migration behaviors: particle rotation is mainly studied in simple shear flows, whereas lateral migration is neglected, and studies on lateral migration mostly focus on spherical particles whose rotational behavior is simple. The aim of this review is to provide a summary of the different works existing in the literature on the inertial migration and the rotational behavior of non-spherical particles with a focus and discussion on the remaining scientific challenges in this field.

## 1. Introduction

Many applications in various fields, such as agriculture, biomedicine, environmental sciences, food technology, and the pharmaceutical industry, demand counting, detecting, sorting, and/or orienting particles in a suspending fluid. The conventional separation processes (settling, centrifugation, membrane filtration…) require large volumes and have an important energetic cost. In the past decade, miniaturized particle separation systems using microfluidic flows have been developed: they allow lowering the sample volumes and the energetic cost and increasing the adaptability to automation and the portability of the devices. These microsystems often include a preliminary stage in which the particles are focused into a tight stream. 

A diverse set of methods has emerged to help to achieve focusing at the microscale [1]: some of them require additional sheath flows [2], while others demand an outer force that can be electric [3], magnetic [4], optical [5], or acoustic [6]. However, the requirement of sheath flows and/or external forces complicates the device fabrication, increases the cost, makes miniaturization difficult, and could also damage biological cells flowing in the device. More recently, inertial focusing techniques have gained significant attention, since they only rely on the behavior of the particles in the channel without using any sheath flows or outer forces. This behavior is controlled by the hydrodynamic interactions between the fluid and the particles and between the different particles flowing together.

**Figure 1:**
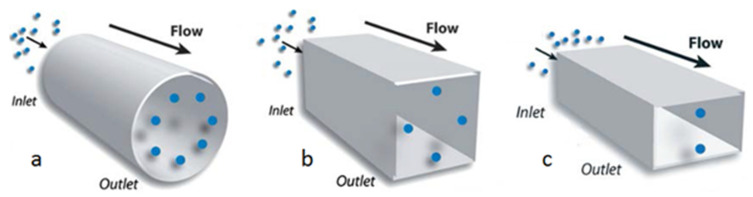
Equilibrium positions at a moderate Reynolds number (in the range 10–250) in a (**a**) circular cross-section (annulus); (**b**) square cross-section (the centers of the four faces); (**c**) rectangular cross-section (the centers of the long faces). Figures (**a**) and (**b**) are republished with permission of Royal Society of Chemistry, from [10]; permission conveyed through Copyright Clearance Center, Inc. The figure (**c**) is republished with permission of Royal Society of Chemistry, from [11]; permission conveyed through Copyright Clearance Center, Inc.

The phenomenon of inertial migration was first observed by Poiseuille in 1836 [7], while studying blood flows; then, it was quantitatively confirmed in 1962 by the experiments of Segré and Silberberg on spherical particles [8,9]. It has been shown that under specific flow and geometrical conditions, flowing particles naturally migrate toward lateral equilibrium positions where they concentrate. The number and the locations of the equilibrium positions vary in function of parameters related to the channel (cross-section (Figure 1) and length), the particle (shape, size and deformability), the suspension (solid volume fraction and type of suspending fluid), and the flow (flow rate, type of flow). After migrating laterally, the particles are shown to form trains, as it will be detailed in Section 3.

**Figure 2:**
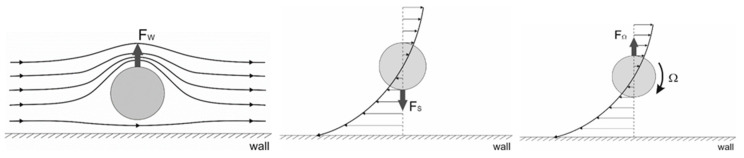
Illustration of the wall-induced lift force *F_w_*, the shear-induced lift force *F_s_*, and the rotation-induced lift force *F_Ω_*.

The mechanisms of inertial migration were highlighted by Matas et al. [12]. Three primary lift forces carry the particles across the streamlines toward their equilibrium positions in rectangular and square microchannels: the wall-induced lift force that pushes the particle away from the wall, the shear-induced lift force that drags the particle close to the wall, and the rotation-induced lift force. These three lift forces are depicted in Figure 2. 

Most of the theoretical, numerical, and experimental works conducted on inertial migration during the last two decades have mainly investigated spherical particles. This simple geometry can be easily fabricated for experimental studies and quickly represented mathematically and numerically. These works have increased the knowledge on the transport of spherical particles in microchannels and demonstrated the ability of inertial focusing methods to separate particles based on their sizes. For example, Di Carlo [13] highlighted the possibility to separate particles of different sizes within the transverse plane of the channel. The separation was experimentally proven by Bhaghat et al. [14] by extracting 590 nm particles from a mixture of 1.9 µm and 590 nm particles using inertial migration.

However, particles found in real suspensions are rarely spherical. For instance, a human red blood cell adopts a biconcave disk-like shape in the body, Escherichia coli is cylindrical, and Euglena gracilis has an ellipsoidal geometry. Furthermore, in many applications, particles of the same size but different geometries can be found, which complicates the size-based separation or even makes it impossible. Thus, shape could serve as a specific marker in the separation, which raises a question: How do non-spherical particles travel in a microchannel? The transport of non-spherical particles in a microchannel is indeed much more complicated than the transport of spherical ones, since their translational and rotational motions are strongly coupled [15,16,17]. Thus, both rotational and migratory behaviors should be considered when studying non-spherical particles flowing in a microchannel. Other particle properties such as deformability or surface topography can play an important role in inertial focusing [18], but the physical mechanisms involved are different. As the main objective of this review was to better understand the role played by the particles’ shape in their inertial migration and rotational behavior, these two parameters were not considered in our analysis. 

The aim of this review is, by analyzing and comparing works in the literature investigating the flow transport of rigid non-spherical particles, to identify and summarize the main findings and remaining questions related to the behavior of complex shape particles flowing in microchannels. Section 2 covers the studies done on the rotational motions of different non-spherical particles in straight shear flows. In Section 3, we present the transport of non-spherical particles flowing in a straight square microchannel where the lateral migration has to be considered. Finally, in Section 4, an overall summary of the review is given, conclusions are drawn, and unanswered questions related to the inertial migration and rotational behavior of a non-spherical particle are identified. Table 1 summarizes the references dealing with suspensions of non-spherical particles cited in this review paper. These works are classified according to the phenomena investigated (rotational behavior, inertial migration), the type of flow (unbounded and bounded shear flows, Poiseuille flow), and the approach adopted by the researchers (theoretical, numerical, or experimental approach). 

## 2. Rotational Behavior of a Non-Spherical Particle in a Shear Flow

**Figure 3:**
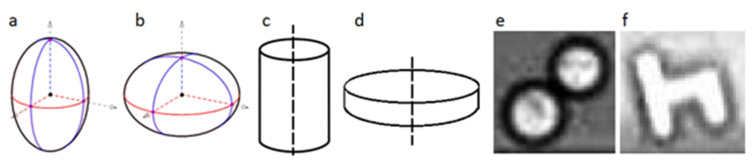
Non-spherical geometries: (**a**) prolate (ellipsoid); (**b**) oblate (ellipsoid); (**c**) cylinder (non-ellipsoidal axisymmetric); (**d**) disc (non-ellipsoidal axisymmetric); (**e**) axisymmetric doublet; (**f**) h-particle (asymmetric); ((**e**,**f**) are reprinted from [55], with the permission of AIP Publishing).

Different geometries have been studied in the literature. They can be classified into three main categories: ellipsoids, non-ellipsoidal axisymmetric particles, and asymmetrical ones (see Figure 3). Based on the aspect ratio of the particle (*λ* = radial diameter/equatorial diameter), an ellipsoid can be a prolate or an oblate (*λ >* 1 and *λ* < 1, respectively). Similarly, non-ellipsoidal axisymmetric particles are subclassified into rod-shaped particles (*λ >* 1) and disc-shaped ones (*λ* < 1). 

### 2.1. Ellipsoidal Particle

Since an ellipsoid is the closest non-spherical shape to a sphere, the first studies done on the effect of the shape of a particle on its rotational behavior investigated ellipsoidal particles. These first studies were mainly theoretical and numerical.

#### 2.1.1. Jeffery’s Theory

In 1922, Jeffery [19] theoretically studied the rotational behavior of an ellipsoid in a simple shear flow. The particle was rigid, isolated, and neutrally buoyant; the fluid was Newtonian, and the suspension flowed in a simple shear, creeping, unbounded flow (i.e., with a constant shear rate and with neglected fluid inertia and wall effects).

**Figure 4:**
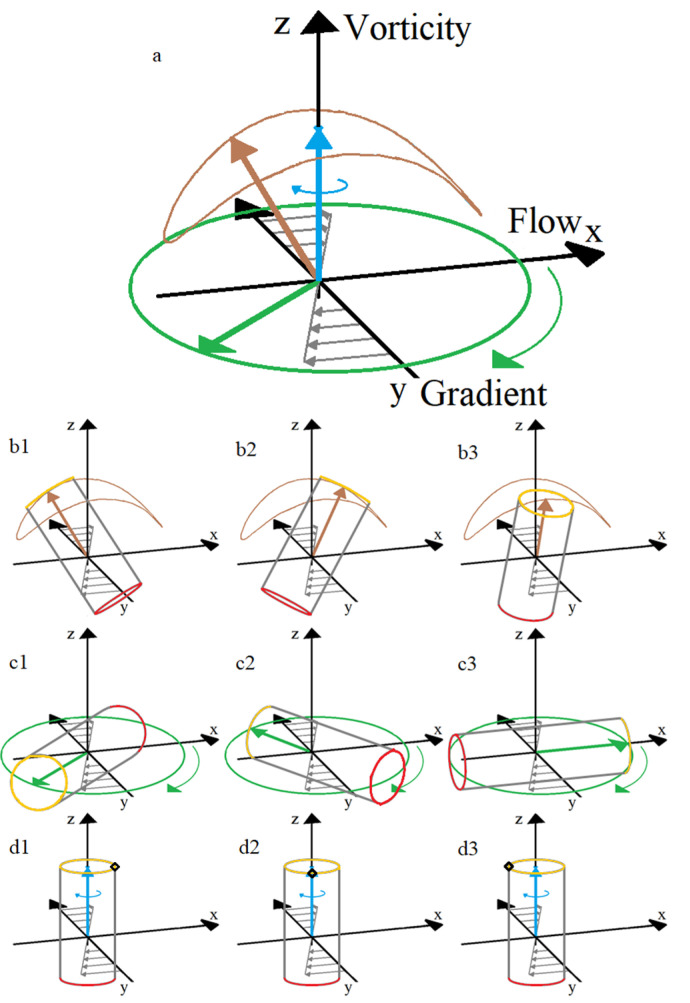
Schematics of Jeffery’s orbits: (**a**) the log-rolling motion (blue), the tumbling motion (green), and the kayaking motion (brown); (**b1**–**b3**): kayaking at three different moments for a cylindrical particle; (**c1**–**c3**): tumbling at three different moments for a cylindrical particle and (**d1**–**d3**): log-rolling at three different moments for a cylindrical particle.

Jeffery found that an ellipsoid rotates around the vorticity axis (perpendicular to the flow-gradient plane) along one of an infinite set of closed orbits, the so-called Jeffery orbits, which are dependent on the initial conditions, the shear rate, and the spheroid’s aspect ratio. This mode of rotation is called “kayaking”, since the particle’s trajectory resembles the motion of a kayak paddle (Figure 4a,b1–b3). The two extreme orbits are called “tumbling”, when the axis of revolution of the particle is rotating in the flow-gradient plane (Figure 4a,c1–c3), and “log-rolling” when the particle rotates around its axis of revolution aligned with the vorticity axis (Figure 4a,d1–d3).

Jeffery also showed that the particle’s angular velocity is not constant but periodic. It has a maximum value when the particle’s axis of revolution is perpendicular to the direction of the fluid motion and a minimum value when the particle is aligned with the flow. The period of rotation *T* is the time needed to complete one orbit. It depends on the shear rate *γ* and the particle’s aspect ratio *λ*, and it can be calculated as follows:

*T* = (*λ* +1/*λ*) × 2*π*/*γ*. (1)

#### 2.1.2. Extension of Jeffery’s Theory for General Shear Flows

Bretherton et al. [20] extended Jeffery’s theory. They theoretically investigated the rotational behavior of an ellipsoid flowing in a general shear flow, where the shear rate *γ* changes with the position. The results validated Jeffery’s analysis: the particle orbits around the vorticity axis and has a period of rotation *T* that can be calculated using Equation (1), with *γ* equal to the shear rate at the center of the studied particle.

#### 2.1.3. Effect of Fluid Inertia

Fluid inertia is shown to have a significant influence on the dynamics of particles suspended in a fluid: it makes ellipsoids adopt a rotational behavior that is different from the one found by Jeffery [19].

For a small non-zero Reynolds number, a prolate particle gradually turns its axis of revolution into the flow-gradient plane in order to tumble [27] (Figure 4a1,a2). The tumbling mode corresponds to the orbit with the maximal energy dissipation [21,28]. At high Reynolds numbers, other rotational modes have been observed by several authors. Ding and Aidun [36] found that above a critical Reynolds number (*Re* ≈ 29 for neutrally buoyant particles), a prolate can cease rotating in a steady-state flow. According to them, this change in the particle’s rotational motion is due to modifications in the recirculation zones created around the moving particle. In the absence of inertia, the streamlines are fully attached, and therefore, the torque on the ellipsoid is always forcing it to rotate, creating a time-periodic state. This rotating state is stable. However, at *Re* > 0, the streamlines detach, creating recirculation zones that influence the particle’s behavior by generating an opposing torque and thus decreasing the net torque exerted on the particle. Beyond the critical Reynolds number, the opposing torque becomes high enough to stop the particle from rotating. This steady-state mode has not been observed by other authors. Qi and Luo [32,33] identified in lieu three different rotational modes with increasing inertia: at low *Re (Re* < 205*)*, the prolate tumbles in the flow-gradient plane; at intermediate *Re* (205 < *Re* < 345), the axis of the spheroid deviates from the flow-gradient plane, and at higher *Re* (345 < *Re* < 467), the prolate rolls with its revolution axis aligned with the vorticity axis. According to Yu et al. [37], a prolate spheroid flowing in a simple shear flow with *Re* increasing from 0 to 256 undergoes the following transitions: Jeffery’s orbit, tumbling, kayaking, log-rolling, and inclined rolling (log-rolling around a tilted axis with respect to the vorticity axis). Huang et al. [38] also found numerically an additional rotational mode that they called inclined kayaking. Both Yu et al. [37] and Huang et al. [38] found that some of these solutions co-exist for a given set of parameters and that the final rotational mode depends on the initial conditions.

**Figure 5:**
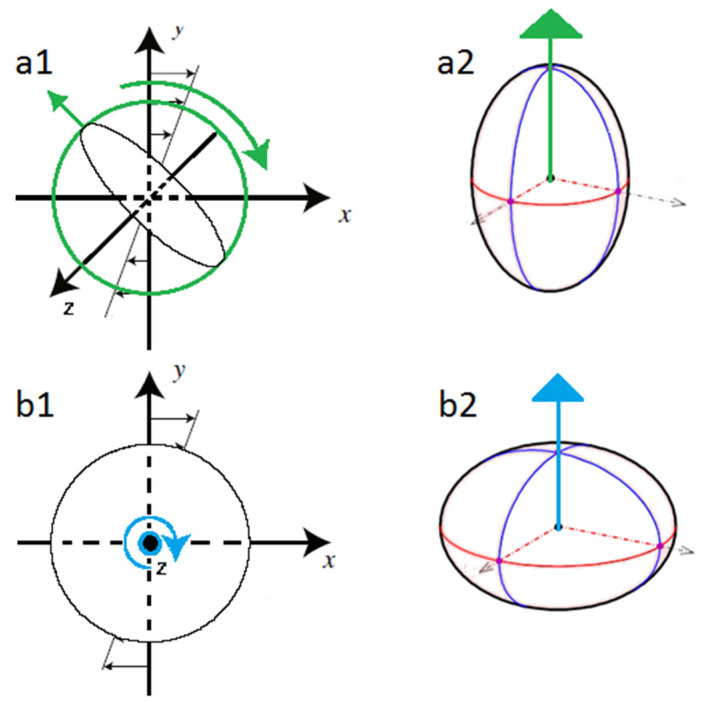
(**a1**) A tumbling prolate; (**a2**) a prolate with its axis of revolution in green; (**b1**) a log-rolling oblate; (**b2**) an oblate with its axis of revolution in blue. Figures (**a1**) and (**b1**) are adapted from [39].

In the case of an oblate and in the presence of fluid inertia, Saffman [27] theoretically showed that the axis of symmetry of the particle gradually aligns with the vorticity axis imposing a log-rolling rotational mode (Figure 5b1,b2). For high Reynolds numbers (220 < *Re* < 467), Qi and Luo [32,33] showed that the inclined log-rolling is the dominant rotational regime. According to Yu et al. [37], an oblate spheroid in a simple shear flow with *Re* increasing from 0 to 192 undergoes the following transitions: Jeffery orbit, log-rolling, inclined rolling, and steady-state. During this last rotational mode, the oblate does not rotate and has its axis of revolution aligned with the gradient axis.

To summarize, the studies converge on the fact that prolate ellipsoids flowing in low-inertia shear flows tumble and oblate particles log-roll. However, at higher values of *Re*, the results diverge, and different rotational modes are observed.

#### 2.1.4. Extension of Jeffery’s Theory for Bounded Flows

In Jeffery’s theory, the particle is flowing in an unbounded flow where no wall effect on the particle is considered. Chwang [22] theoretically studied the rotational behavior of ellipsoids in bounded Couette and Poiseuille flows and showed that Jeffery’s results were applicable as long as the particle is far from the walls.

#### 2.1.5. Effect of Buoyancy (Sometimes Called “Particle Inertia”)

Jeffery’s theory [19] is based on a neutrally buoyant condition, where the particle and the fluid have the same density. While increasing the particle-to-fluid density ratio, Ding and Aidun [36] observed that the ellipsoid’s rotational modes are conserved and that only the transition Reynolds numbers between the different modes are increased.

#### 2.1.6. Effect of the Particle’s Aspect Ratio

In the absence of fluid inertia, Equation (1) shows that an increase in the particle’s aspect ratio causes an increase in the rotation period. This phenomenon has been shown to be still valid in the presence of fluid inertia in [40]. 

Moreover, the variation in the rotational rate is shown to increase when the ellipsoid’s aspect ratio is increased: the maximum angular velocity (when the particle is perpendicular to the flow) becomes higher, and the minimum angular velocity (when the particle is aligned with the flow) becomes lower. This was also reported by Bretherton [20], who observed that an elongated prolate with a high value of *λ* spends most of its time almost parallel to the streamlines, and it reverses itself periodically. 

#### 2.1.7. Effect of the Particle’s Initial Orientation

In the absence of inertia, Jeffery’s theory [19] considered that the particle’s initial orientation has a determinant effect on its trajectory and the choice of the orbit. When the inertial effects start taking place, the conclusions are more controversial. Yu et al. [37] showed theoretically that the trajectories of prolates and oblates flowing in a Couette flow at a given Reynolds number are determined by their initial orientations. However, the results of Huang et al. [38] and Rosén et al. [41] indicate that the rotational mode of an ellipsoid is influenced by its initial orientation only within certain intervals of *Re*. 

### 2.2. Axisymmetric Non-Ellipsoidal Particle

Due to several challenges in the fabrication of ellipsoids for experimental testing and since the non-spherical particles that flow in real suspensions have more complex shapes, some of the studies were extended from ellipsoids to axisymmetric non-ellipsoidal particles (mainly rods, doublets, and discs). These works helped understand the effect of the shape of the particle on its behavior, especially when the particle has sharp edges and/or is more flattened or elongated than an ellipsoidal particle.

#### 2.2.1. Extension of Jeffery’s Theory to Axisymmetric Non-Ellipsoidal Particles

Bretherton [20] showed that Jeffery’s equations are also valid for almost any axisymmetric particle, provided that an equivalent aspect ratio *λ_e_* is used. This latter is determined experimentally by measuring the orbital period [20,50]. So, it can be deduced that an axisymmetric non-ellipsoidal neutrally buoyant particle in simple, creeping, and unbounded flows kayaks around the vorticity axis in a closed orbit. The orbit is determined by the particle’s initial orientation, and the rotational period depends on the equivalent aspect ratio.

#### 2.2.2. Effect of Fluid Inertia

As seen previously, in the presence of inertia, a prolate particle gradually turns into the flow-gradient plane in order to tumble, whereas an oblate particle gradually aligns its axis with the vorticity axis in order to log-roll. With increasing Reynolds number, other rotational modes can appear.

In the same way, in the presence of fluid inertia, a rod-like particle (*λ_e_* > 1) tumbles in the flow-gradient plane, while a disc-like particle (*λ_e_* < 1) rolls with its axis of revolution perpendicular to this same plane [29,30,51,52]. A Reynolds number *Re* ~O(10^−3^) is sufficient to force rods and discs to drift from kayaking to a new rotational behavior [23,24,29]. 

Ku and Lin [42] obtained similar results for fibers that are rod-like particles with a large aspect ratio. At *Re* = 0, a fiber in a simple shear flow far from the walls rotates with a constant period that can be accurately calculated using Jeffery’s equations. At weak inertia, the fiber still rotates periodically, but it slowly drifts toward the flow direction. Similar to the case of prolates and rod-like particles, low inertia forces the fiber to tumble in the shearing plane perpendicular to the vorticity.

Above a critical Reynolds number (4 < *Re_c_* < 13, varying with the particle’s aspect and confinement ratios), Zettner and Yoda [53] showed experimentally that a cylindrical particle can cease rotating, resting at a nearly horizontal equilibrium orientation. Ku and Lin [42] obtained similar results for fibers, for a particle Reynolds number *Re_p_* ≥ 6 (*Re_p_* is the Reynolds number based on the length of the particle and the flow average velocity). In addition, they showed that the stable stationary orientation is established with the fiber’s axis tilted by a small angle about the flow direction.

#### 2.2.3. Walls and Confinement Effects

The experimental study of Poe and Acrivos [54] and the numerical simulations of Aidun [43] on the transport of rods in simple shear flows revealed that low confinement ratios (*κ* < 0.32) have negligible effects on the particle’s behavior. This parameter, also called blockage ratio, is the ratio between the diameter of the particle and the smallest dimension in the channel cross-section. However, *κ* has a marked effect at higher values [36,53].

Increasing the confinement degree causes an increase in the value of the critical Reynolds number above which a fiber changes its rotational behavior from a periodic tumbling to a steady state, meaning that it delays the transition from time-periodic rotation to stationary state. It also decreases the angle between the fiber stationary equilibrium orientation and the streamwise direction [42]. However, for a highly confined flow, a fiber has a stable stationary orientation through the whole range of Reynolds numbers studied in this paper.

#### 2.2.4. Effect of the Particle’s Aspect Ratio 

As explained previously, an increase in the aspect ratio of an ellipsoid increases its period of rotation and can also alter its rotational behavior. 

**Figure 6:**
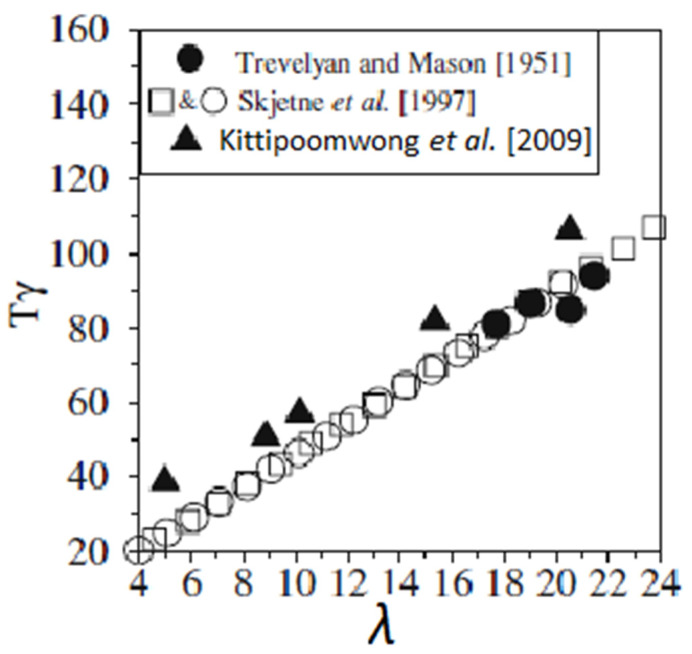
Normalized period of rotation as a function of the aspect ratio of a cylindrical rod (reprinted from [34] by permission from Springer Nature Customer Service Centre GmbH).

Similar results were obtained in the case of cylinders. Trevelyan and Mason [31], Kittipoomwong et al. [34], and Skjetne et al. [44] showed that when the aspect ratio of a cylinder *λ* increases at a constant shear rate, the cylinder’s angular velocity decreases and its period of rotation increases (Figure 6). However, the measured period of rotation was less than the one predicted by Jeffery [19] when the actual aspect ratio *λ* was inserted in Jeffery’s equations. For example, Trevelyan and Mason [31] observed that the period of rotation of a cylindrical particle is equal to two-thirds the period of rotation of a prolate with the same aspect ratio. This deviation is due to the non-spheroidal form and can be taken into consideration by replacing the aspect ratio *λ* by the equivalent ellipsoidal axis ratio *λ_e_* [20,29,31,50,59]. For rod-like particles, it was found experimentally and numerically that *λ_e_*/*λ* ≈ 0.7 [31,35].

An increase in the particle’s aspect ratio also modifies the rotational mode. It increases the critical Reynolds number above which the particle adopts a steady state, i.e., stops rotating [53]. In addition, the maximum value of the angular velocity (when the particle is perpendicular to the flow) increases, and its minimum value (when the particle is aligned with the flow) decreases.

#### 2.2.5. Effect of the Solid Volume Fraction (Suspension’s Concentration) 

Anczurowski et al. [51,52] showed that the drift from Jeffery’s orbits to a tumbling motion occurred faster (i.e., at a smaller *Re*) by increasing the suspension’s concentration. 

Table 2 summarizes the key conclusions regarding the rotational behavior of ellipsoidal and axisymmetric non-ellipsoidal particles in shear flows.

### 2.3. Asymmetric Particles

On a higher level of complexity, few works investigated the effect of the particle asymmetry on its rotational behavior in a shear flow. Gierszewski and Chaffey [25] ran numerical simulations to study the rotation of a triaxial ellipsoid in a simple shear flow. They showed that if the particle is asymmetric, its motion is qualitatively different from that of an axisymmetric particle, and it has no fixed period of rotation around the fluid’s vorticity axis. In fact, once the symmetry is broken, the period of rotation is altered, and the more the particle is asymmetric, the more the period is modified. As a function of the asymmetry in the particle’s geometry, particles can tumble periodically, quasi-periodically (also called doubly periodic), or in a chaotic way [26]. The behavior can be chaotic in both space and time references.

## 3. Rotational and Migratory Behaviors of a Particle Flowing in a Square Microchannel

In this section, we present several studies investigating the behavior of a particle in a square microchannel and the effect of parameters related to the particle, the channel, the suspension, and the flow on this behavior. 

### 3.1. Spherical Particles

As seen in the introduction, neutrally buoyant spherical particles flowing at moderate Reynolds numbers in a square microchannel laterally migrate toward four equilibrium positions located at the centers of the channel walls. The lateral migration consists of two main stages: the particle migrates first in the lateral direction through the velocity iso-contours and reaches an equilibrium ring; then, it moves cross-laterally along the chosen ring toward its equilibrium position [12,60,61,62,63]. In the first stage, called the cross-streamline migration, the shear-induced and the wall-induced lift forces drive the particles to a rectangular ring-like region where the effects of both forces are canceled. The rotation-induced lift force dominates in the second stage of migration (called the cross-lateral migration) and drives the particles to their equilibrium positions with a smaller migration velocity.

Depending on the Reynolds numbers, three different migration regimes can be observed. At low *Re* (*Re* < 5 in the experimental conditions of [60]), spheres laterally migrate toward the center of the channel. At moderate *Re* (5 < *Re* < 200), particles migrate toward the four equilibrium positions described above. At high Reynolds numbers (*Re* > 450, according to [62]), secondary equilibrium positions appear at the corners of the channel, increasing the total number of equilibrium positions from four to eight. Regardless of the regime, increasing the Reynolds number accelerates the migration process and thus decreases the focusing length. The distance between a face-centered equilibrium position and its nearest wall varies with the Reynolds number. An increase in *Re* in the moderate range gets the spheres closer to the walls [60]. However, at high values of *Re*, increasing this number increases the distance between the face-centered equilibrium positions and the walls, and the particles are observed to be closer to the channel centerline [62].

The confinement ratio *κ* is also shown to have an effect on the sphere’s inertial migration. In the case of neutrally buoyant particles, increasing *κ* accelerates the migration process and thus decreases the channel length needed to reach the final stage of migration. In addition, bigger spheres (i.e., higher *κ*) focus at an equilibrium position closer to the channel centerline than smaller particles [60].

**Figure 7:**
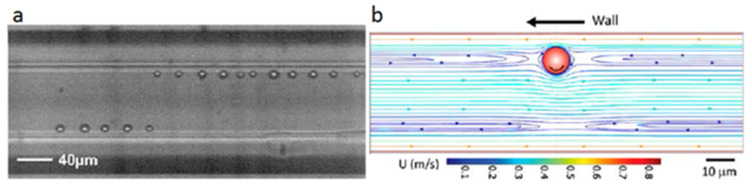
(**a**) A captured image representing the longitudinal ordering of spherical particles in a square channel at *Re* = 210 (reprinted from [63]; (**b**) Simulated streamlines around an isolated sphere at *Re* = 48 putting into evidence the reversing streamlines created at both sides of the particle (reprinted from [61]).

The migration toward the equilibrium positions is followed by a particle longitudinal ordering, during which the particles form trains and follow each other in the channel with a constant interparticle spacing (Figure 7a). This phenomenon only occurs after the lateral migration is fully developed. This was observed by Segré and Silberberg [8,9]; then, it was investigated and explained by Matas et al. [64] and Lee et al. [61]. The “reversing streamlines” created around the particle while flowing in the channel were found to be the physical phenomenon underlying this particle alignment (Figure 7b).

**Figure 8:**
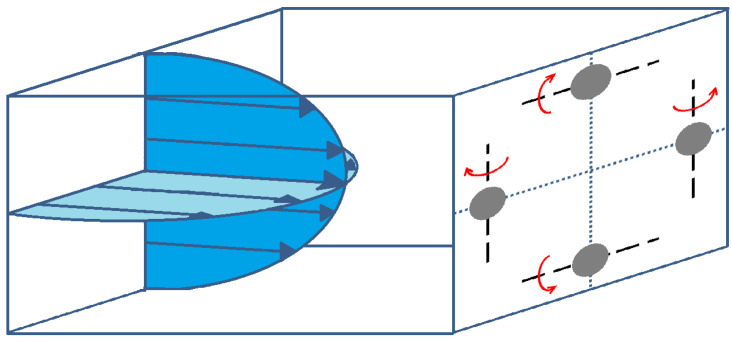
A schematic representing a square microchannel in which particles are focused on their equilibrium positions and are rotating around their rotational axis (dashed black lines).

In addition to their inertial migration, particles have a rotational behavior in the channel. According to [60], the rotational behavior of a spherical neutrally buoyant particle flowing in a square microchannel at moderate Reynolds numbers depends on the sphere’s lateral migration development. During the migration process, the particle’s angular velocity has three components along the three main axes of the channel: it rotates around both cross-sectional directions due to the presence of two parabolic velocity profiles in a typical square Poiseuille flow, and it rotates also around the flow axis. Once the particle is focused, only the rotation in the cross-sectional direction parallel to its closest wall remains. The particle’s angular velocity increases with increasing *Re*. Figure 8 shows particles focused on their equilibrium positions in a square cross-section with their rotational axis.

### 3.2. Non-Spherical Particles

#### 3.2.1. Rotation Behavior and Lateral Migration at Moderate Reynolds Numbers 

The main difficulty in the study of non-spherical particles is that their migration is strongly coupled with their orientation and rotational regime [17]. For example, the lift component induced by the particle’s rotation has an obvious influence on the transverse focusing position of the particles, which cannot be neglected [65].

**Figure 9:**
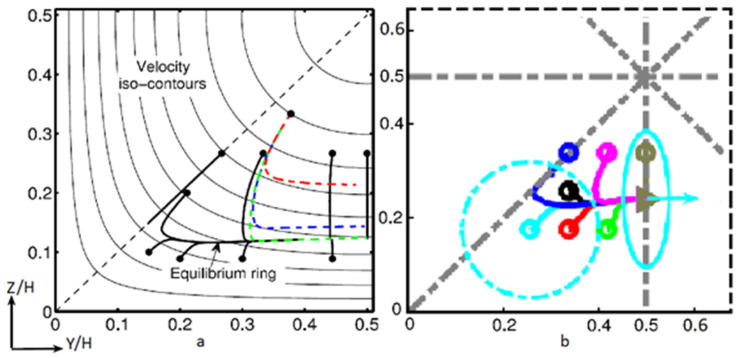
Stages of inertial lateral migration of: (**a**) a sphere. The solid circles are the initial positions of each trajectory. The thick black lines are the projection of particle trajectories for *Re* = 120. The dark, mid, and light gray dashed lines (red, blue, and green) are the respective trajectories for *Re* = 12, 60, and 120 launched from the same initial position (reprinted from [60], with the permission of AIP Publishing); (**b**) an oblate at *Re* = 100. The open circles and triangles show the initial position and final equilibria, respectively. The initial and final orientations of the oblate are shown for a particular case with the light blue solid line (reproduced from [16] with permission).

In 2017, Lashgari et al. [16] were the first to study the motion of oblate particles in a microfluidic configuration where not only the equilibrium position but also the entire migration dynamics of the particle from the initial to the final position, including particle trajectory, velocity, rotation, and orientation, were investigated. The results of their numerical simulations reveal phenomena similar to those observed for spherical particles. In particular, an oblate particle experiences at moderate *Re* a lateral motion toward a face-centered equilibrium position so that four face-centered equilibrium positions are observed as for spheres (Figure 9). Likewise, oblates migrate in a two-stage process: they first migrate laterally toward a square annulus in the vicinity of the walls, and they secondly move cross-laterally in the annulus toward the four-centered equilibrium positions. 

More recently, Nizkaya et al. [17] studied inertial focusing of oblate spheroidal particles in channel flow at moderate Reynolds numbers *Re* = 11–44 using lattice Boltzmann simulations. They found also that all spheroids focus on the four face-centered equilibrium positions.

For cylindrical particles flowing in a square channel as well, Su et al. [45] demonstrated numerically that there are always four stable equilibrium positions for *Re* varying from 50 to 200. The two-stage process (cross-streamline and cross-lateral migrations) was also shown for these cylindrical particles.

**Figure 10:**
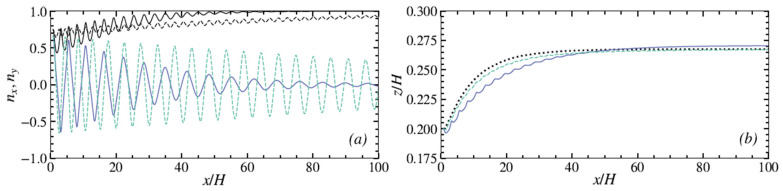
(**a**) *x*-components (colored curves) and *y*-components (black curves) of the orientation vector of an oblate and (**b**) trajectories for spheroids with *κ* = 0.15 and *λ* = 0.5 (solid), 0.8 (dashed), and 1 (dotted) (reprinted from [17], with the permission of AIP Publishing).

While the particles migrate toward their equilibrium positions, their rotational mode changes. Pan et al. [46] showed that an ellipsoidal particle in a Poiseuille flow has a stable orientational behavior after reaching its equilibrium position: a prolate ellipsoid tumbles and an oblate ellipsoid log-rolls, as they do in a shear flow at moderate *Re* (see Section 2). According to [16,17], the streamwise motion of an oblate spheroid, from its initial to its final equilibrium position is coupled to a rotation around its axis of revolution (log-rolling) and a tumbling around its equatorial axis creating the already described kayaking mode. As the particle gets closer to the equilibrium position, the tumbling motion vanishes gradually, and the particle just rotates around its axis of revolution (Figure 10). This means that oblates start to migrate with a kayaking motion and slowly move to a log-rolling motion while getting closer to their equilibrium lateral positions. The kayaking motion is responsible for oscillations of the trajectories.

Lashgari et al. [16] found that, on its final equilibrium position, the orientation vector of an oblate particle is parallel to the nearest wall, perpendicular to the flow. This is in agreement with the experimental findings of Di Carlo et al. [13] on the inertial focusing of particles in microchannels. Finally, at the equilibrium position, an oblate particle rotates around its axis of revolution (with zero streamwise and wall-normal rotation rates).

According to [16], the focusing length in square channels is slightly longer for oblate particles compared to spherical particles of the same volume starting from the same initial lateral position. This is mainly attributed to the presence of a tumbling motion during the migration, which reduces the lateral velocity of the oblates. However, this is not the case in rectangular channels where the focusing length is slightly shorter for oblates than for spheres. Tumbling is negligible in that case, and it has been numerically found that the lateral velocity for oblate particles is higher than that of spheres in a rectangular cross-section [16]. 

**Figure 11:**
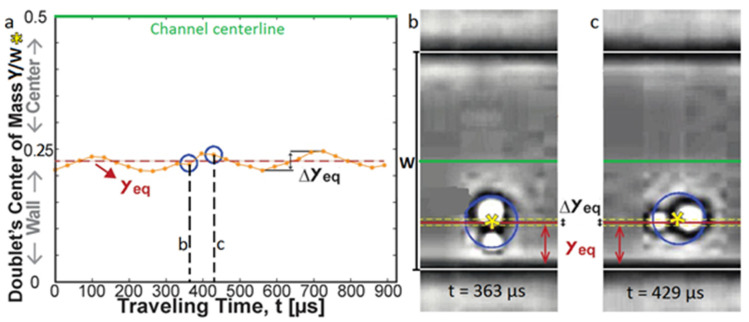
The oscillations of the center of mass of a doublet around its equilibrium position *Y_eq_*. This latter is determined by averaging the values of the positions of the particle’s center. Blue circles in (**a**) indicate the data points retrieved from the high-speed microscopic images (**b**,**c**) taken at the corresponding times (reprinted from [55], with the permission of AIP Publishing).

Unlike spherical particles [16,60], non-spherical ones oscillate while being transported in a microchannel flow. Hur et al. [55], Masaeli et al. [49], and Su et al. [45] observed that an elongated particle (*λ* > 1) will keep oscillating regardless of its position in the channel (still migrating or focused on an equilibrium position) (Figure 11). This might be due to the fact that a prolate particle prefers to tumble (see Section 2.1.3) and that, while adopting this type of rotation, the angular velocity, the sweeping area, and the distance from the wall continuously change, leading to periodical variations of the wall-induced lift force and thus a periodic motion of the particle toward and away from the wall [45]. In the same way, Nizkaya et al. [17] showed that an oblate particle oscillates while kayaking, since the kayaking motion is a combination of tumbling and log-rolling but stopped oscillating once it is focused on its equilibrium position.

Despite this oscillatory behavior, the magnitudes of the observed oscillations are very small: around 1.25% of the particle’s length [45]. It can be deduced that similarly to spheres, non-spherical particles have well-defined equilibrium positions.

#### 3.2.2. Influence of the Reynolds Number *Re*

Similar to spherical particles, Hur et al. [55] experimentally showed that neutrally buoyant discs, rods, and doublets migrate toward the channel center at low Reynolds numbers (*Re* < 14). 

**Figure 12:**
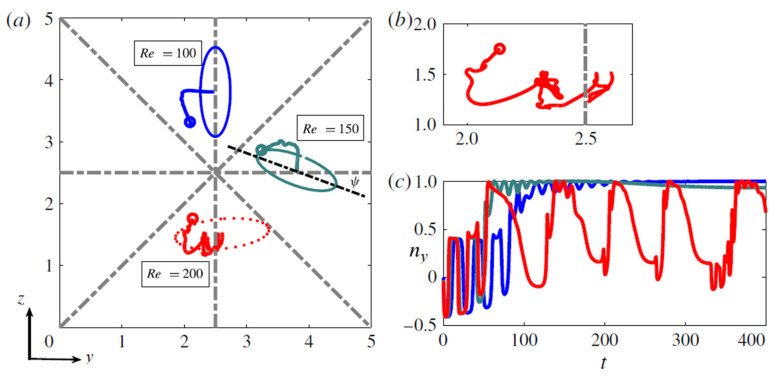
(**a**) Lateral trajectories of a rigid oblate particle in a square channel at different Reynolds numbers. Open circles and triangles show the initial and final equilibrium positions, respectively. The final orientation of the oblate spheroid is shown with solid and dashed (for unstable cases) lines. (**b**) Zoom on a particle trajectory at *Re* = 200. (**c**) The *x*-component of the orientation vector, *n_x_*, as a function of time for the different Reynolds numbers under investigations (same color coding as in panel a) (reproduced from [16] with permission).

For an oblate particle flowing in a square microchannel at moderate and high Reynolds numbers, Lashgari et al. [16] identified three migration regimes, as seen in Figure 12:

- For *Re* < 150, particles log-roll and migrate toward the four face-centered equilibrium positions, with the particles being closer to the wall with increasing *Re*;
- For 150 < *Re* < 200, an oblate particle still focuses close to a face-centered equilibrium position but with its axis not perpendicular to the closest wall. This rotational mode resembles the inclined rolling mode reported in the dynamical system analysis of the motion of an oblate spheroid in a simple shear flow [36,39]. In that range of *Re*, particles still get closer to the wall when *Re* increases;
- For *Re* > 200, an oblate particle approaches one of the four standard equilibrium positions, but its orientation and rotation are time-dependent and chaotic. The rotational mode seems to be a combination of the tumbling and the log-rolling motions. This behavior of oblates was also observed by Rosén et al. [39], who showed that above a certain particle Reynolds number, the tumbling motion can be found in addition to the already existing log-rolling mode. At *Re* > 300, the particle focuses closer to the channel center.

Similar results have been obtained for rod-like particles. In a square channel, while increasing the fluid inertia, Su et al. [45] (*Re* = 50–400) did not observe any modification in the distribution of cylindrical particles in the channel. The non-spherical particles kept their four equilibrium positions at the centers of the channel walls regardless of *Re*. In addition, no works showed the transition from four to eight equilibrium positions, which was observed for spherical particles (see Section 3.1). 

Moreover, Masaeli et al. [49] observed that at a fixed distance from the channel inlet, the percentage of tumbling prolate particles increased from 39% at *Re* ≈8 to 84% at *Re* ≈ 20. So, an increase in the Reynolds number increases the probability of finding a prolate particle with a tumbling rotational mode. It might be deduced from this observation that an increase in *Re* accelerates the transition phase from the kayaking mode to other modes (tumbling for prolates and log-rolling for oblates), lowering by that the channel length needed to reach the equilibrium rotational mode. Note that the Reynolds number has the same effect on the focusing length: when *Re* increases, the equilibrium positions are reached faster [55].

**Figure 13:**
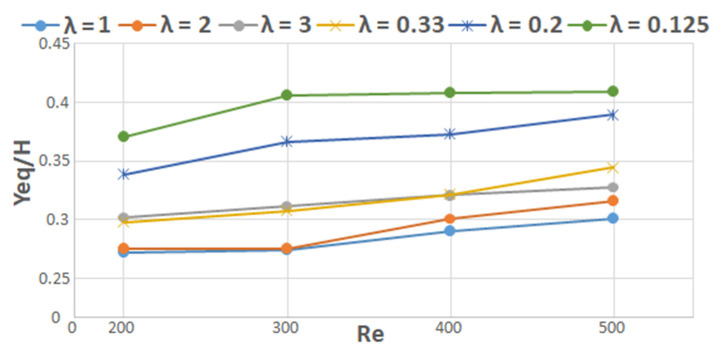
The results of Huang et al. [47] represented as the evolution of the normalized equilibrium position of an ellipsoidal particle (prolate and oblate) as a function of the Reynolds number. *H* is the channel’s height.

Finally, it has been observed that the effect of the Reynolds number on the distance between the equilibrium positions of spheres and the closest wall is the same in the case of non-spherical particles. The experimental results in [49] showed that the equilibrium positions of rod-like particles move closer to the channel wall while increasing the Reynolds number up to 200. The numerical simulations of Huang et al. [47] showed also that the focusing positions of oblate particles tend to get closer to the channel centerline while increasing *Re* above 200 (Figure 13).

#### 3.2.3. Influence of the Confinement Ratio *κ*

At moderate Reynolds numbers, when spherical particles migrate toward the four face-centered equilibrium positions, it is seen that spheres get closer to the centerline when the confinement degree increases.

**Figure 14:**
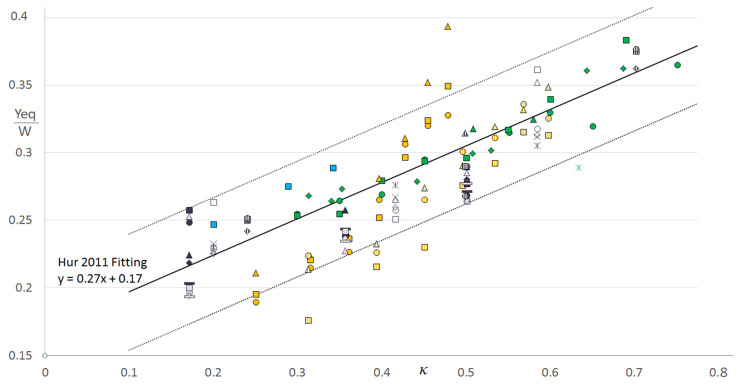
The evolution of the normalized equilibrium position *Y_eq_/W* as a function of the confinement ratio *κ* (the normalized rotational diameter *d_rot_/W)*. The results are obtained by Lashgari et al., 2017 [16] (blue) [Oblate at *Re* = 100 with *AR_c_* = 0.5 (channel aspect ratio) (filled square)], Hur et al., 2011 [55] (green) [Sphere at *Re* = 200 with 0.6 < *AR_c_* < 0.66 (filled square), Disc at *Re* = 200 with 0.6 < *AR_c_* < 0.66 (filled triangle), Cylinder at *Re* = 200 with 0.6 < *AR_c_* < 0.66 (filled rhombus), Doublet at *Re* = 200 with 0.6 < *AR_c_* < 0.66 (filled circle), Asymmetric disc at *Re* = 200 with 0.6 < *AR_c_* < 0.66 (asterisk)], Li et al., 2017 [57] (yellow) [*E. gracilis* at *Re* = 205 with *AR_c_* = 0.55 (filled square), *E. gracilis* at *Re* = 128 with *AR_c_* = 0.55 (filled circle), *E. gracilis* at *Re* = 77 with *AR_c_* = 0.55 (filled triangle), *E. gracilis* at *Re* = 205 with *AR_c_* = 0.45 (dashed square), *E. gracilis* at *Re* = 128 with *AR_c_* = 0.45 (dashed circle), *E. gracilis* at *Re* = 77 with *AR_c_* = 0.45 (dashed triangle)] and Masaeli et al., 2012 [49] (purple) (Sphere/Rod at *Re* = 13.09 with *AR_c_* = 0.74 (filled triangle), Sphere/Rod at *Re* = 19.64 with *AR_c_* = 0.74 (filled square), Sphere/Rod at *Re* = 26.18 with *AR_c_* = 0.74 (filled rhombus), Sphere/Rod at *Re* = 32.73 with *AR_c_* = 0.74 (filled circle), Sphere/Rod at *Re* = 39.27 with *AR_c_* = 0.74 (dotted triangle), Sphere/Rod at *Re* = 45.82 with *AR_c_* = 0.74 (filled rectangle), Sphere/Rod at *Re* = 52.36 with *AR_c_* = 0.74 (dotted square), Sphere/Rod at *Re* = 58.91 with *AR_c_* = 0.74 (dotted rhombus), Sphere/Rod at *Re* = 65.45 with *AR_c_* = 0.74 (dotted rectangle), Sphere/Rod at *Re* = 72 with *AR_c_* = 0.74 (dotted circle), Sphere/Rod at *Re* = 14 with *AR_c_* = 0.64 (empty triangle), Sphere/Rod at *Re* = 21 with *AR_c_* = 0.64 (empty square), Sphere/Rod at *Re* = 28 with *AR_c_* = 0.64 (empty rhombus), Sphere/Rod at *Re* = 35 with *AR_c_* = 0.64 (empty circle), Sphere/Rod at *Re* = 42 with *AR_c_* = 0.64 (X), Sphere/Rod at *Re* = 49 with *AR_c_* = 0.64 (asterisk), Sphere/Rod at *Re* = 14.8 with *AR_c_* = 0.53 (dashed triangle), Sphere/Rod at *Re* = 22.2 with *AR_c_* = 0.53 (dashed rectangle), Sphere/Rod at *Re* = 29.6 with *AR_c_* = 0.53 (dashed rhombus), Sphere/Rod at *Re* = 37 with *AR_c_* = 0.53 (dashed circle)). The black line corresponds to the fitting curve obtained by the experiments of [55], and the two dashed black lines bound the range of acceptable deviation also called the confidence zone (half of the radius of the smallest particle).

An “equivalent diameter” definition is needed to quantify the confinement for particles of different shapes. The definitions of this diameter vary among the different studies. Hur et al. [55] experimentally observed that the particle’s equilibrium position *Y_eq_* is determined by its rotational diameter *d_rot_* (the longest distance between two points on the particle, which corresponds to the particle’s longest axis of symmetry for most of the cases), regardless of its geometrical shape. This was shown to be true for all the tested geometries (spheres, cylinders, discs, symmetric and asymmetric doublets) except for the case of asymmetric disks (h-particles). The observations of [55] were later numerically confirmed by Masaeli et al. [49]. Thus, we chose the rotational diameter as an equivalent diameter. Figure 14 represents the equilibrium distance from the nearest wall, which is normalized by the channel smallest dimension *W*) as a function of the confinement ratio.

It can be deduced from Figure 14 that the normalized equilibrium position increases with the confinement ratio, which means that non-spherical particles get closer to the channel centerline, as do spheres in the same conditions [63,66]. 

Moreover, the evolution is quite linear, following the fitting curve obtained by Hur et al. [55]. This linear progression was also observed in the case of spherical particles [67,68]. We have added in Figure 14 two parallel lines bounding a confidence zone in which the deviation from the fitting line is considered acceptable. The lines are located at ±0.043 *W* from the fitting curve. This value is equal to the half of the radius of the smallest particle of the data presented in Figure 14 (*d_rot-(min)_/W* = 0.17142). Most of the particles (94%) are within the confidence zone. Solely the results of Li et al. [57] seem to be far from the others, and this deviation could be due to the fact that their work is focused on living microorganisms for which additional parameters such as their deformability can play a role in their displacement in the channel. 

Concerning the effect of the confinement degree on the focusing length, Lashgari et al. [16] numerically found that the focusing length of an oblate particle decreases with increasing *κ*. In other words, large particles focus faster than smaller ones. This is similar to the spherical case where smaller particles need longer channels to reach their focusing positions.

**Figure 15:**
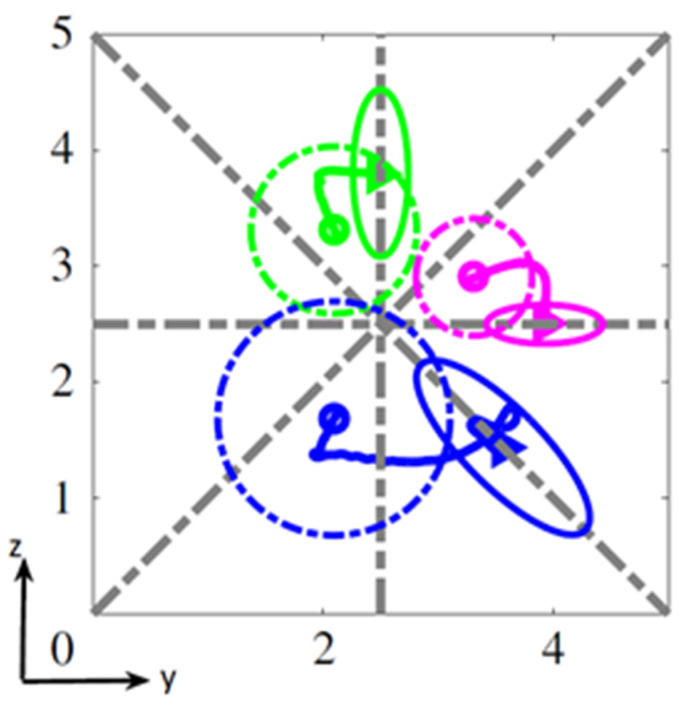
Lateral trajectories of oblate rigid particles of different sizes in a square duct at *Re* = 100. The particles have the same aspect ratio (ratio between the particle axes, *λ* = 1/3) and different volumes. The confinement ratios *κ* are equal to 1/6.93, 1/3.47, and 1/2.43 for the pink, green, and blue particles, respectively. The values of *κ* are calculated using the particles aspect ratio and the equivalent volume diameters *d_v_* (diameter of the sphere of the same volume). Open circles and triangles show the initial and final equilibrium positions, respectively. The initial and final orientations of the oblates are shown with dashed and solid lines (reproduced from [16] with permission).

Finally, Lashgari et al. [16] observed that in a square channel and for a high value of confinement (*κ* = 1/2.43), the migration dynamics of an oblate are completely different. The equilibrium position is along the diagonal symmetry line, between the corner and the center of the channel (Figure 15). In order to explain the peculiar behavior of the large oblate in a square channel, the authors examine the rotation rate of the oblate around the streamwise direction during the migration process. Typically, during the migration stages, the angular velocity increases and then decreases. It increases due to the fast motion of the particle toward the equilibrium wall and then decreases due to the particle’s slow motion toward the equilibrium position under the action of the rotation-induced lift force. The magnitude of this force is reduced when the angular velocity decreases, and the particle eventually focuses at a face-centered equilibrium position. This behavior is not observed for the largest oblates presented here. In this case, the streamwise rotation does not reduce to zero as the particle moves toward the vertical symmetry line, which accelerates the particle motion. This might occur because large oblates are more susceptible to tumbling. As a result of this acceleration, the particle crosses the vertical symmetry line and focuses on the diagonal where the streamwise rotation rate becomes zero and the shear-induced and wall-induced lift forces balance each other. This strange behavior was not observed by Hur et al. [55], who studied the inertial migration in highly confined rectangular channels and confirmed that the particles focus at the centers of the longest channel sides similarly to the cases of low *κ* values.

#### 3.2.4. Influence of the Particle’s Aspect Ratio *λ*

Figure 9a,b, reprinted from Nizkaya et al. [17], present respectively the streamwise component (colored curves) and the vorticity component (black curves) of the orientation vector as well as the trajectories of three oblate particles with the same rotational diameter but different aspect ratios (*λ_1_* = 0.5 (solid), *λ_2_* = 0.8 (dashed), and *λ_3_* = 1 (sphere) (dotted)). As seen in Figure 10a, the streamwise component of the orientation vector experiences decaying oscillations, while the vorticity component converges to unity. This confirms that oblates start with a kayaking motion (responsible for oscillations) and slowly converge to a log-rolling motion with the revolution and rotational axes aligned with the vorticity direction. As seen in Figure 10b, the equilibrium positions for the three oblates are very close, indicating that the equilibrium positions and the log-rolling motions are controlled by the particle’s rotational diameter with a weak dependence on the aspect ratio. This result is in full agreement with the experimental findings of [49,55].

Concerning the rotational behavior, it can be observed that the oscillations vary with the value of *λ*, showing that the particle’s aspect ratio influences the kayaking motion. For example, the oscillations of the less-oblate spheroid (*λ* = 0.8) decay much slower than those for *λ* = 0.5 (more oblate). Moreover, it can be noted that in the case studied by Nizkaya et al. [17], the particles reach their equilibrium position (Figure 10b) before the stabilization of their rotational mode (Figure 10a). This phenomenon is more pronounced when the particle aspect ratio *λ* is closer to 1 (spherical case). 

**Figure 16:**
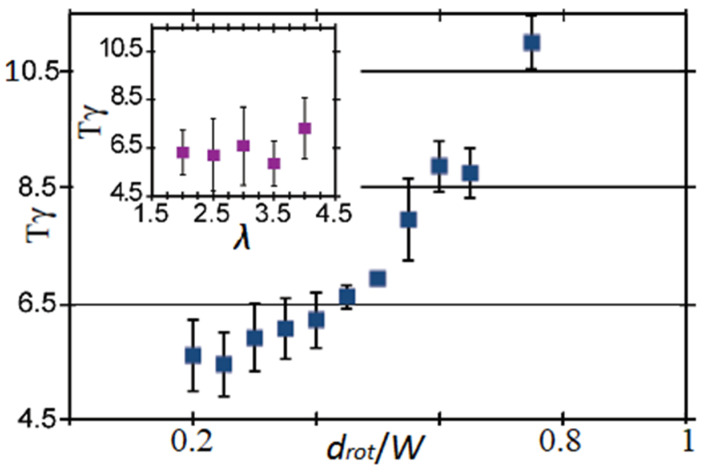
Normalized period of rotation of doublets with various size and asymmetry as a function of the aspect ratio *λ* (purple) and the confinement ratio *d_rot_*/*W* (blue) (reprinted from [55], with the permission of AIP Publishing).

Equation (1), proposed by Jeffery for the calculation of the period of rotation, indicates that an increase in the aspect ratio of the ellipsoid increases its period of rotation *T*. This was shown to be true by Masaeli et al. [49], who studied the behavior of elongated particles (*λ* = 3 and 5) in a rectangular Poiseuille flow. However, this result was not obtained by Hur et al. [55], who claimed that *T* is independent of *λ* and that it increases with the confinement ratio *κ* (Figure 16). However, the study presented in [55] was performed on a small range of *λ* (2 < *λ* < 4). In our opinion, the work should be done on a larger interval in order to validate the obtained results. 

#### 3.2.5. Effect of Other Parameters

Other parameters, such as the particle asymmetry and its initial position and orientation, have been shown to have an influence on the rotational behavior, but the relative literature summarized hereafter is still poor regarding confined flow. 

Effect of asymmetry in the particle’s geometry: In the case of a shear flow (cf. Section 2.3), asymmetry in the particle’s geometry generates chaos in its rotational behavior [25,26]. This was confirmed by Einarsson et al. in a Poiseuille flow [48,56]. However, the findings of Hur et al. [55] in a rectangular channel show that h-shaped particles log-roll similar to axisymmetric discs, and no chaotic behavior is observed.

Effect of the particle’s initial conditions: As shown earlier, in a shear flow, when inertia is present, the effect of the particle’s initial conditions is still unclear. In a Poiseuille flow, with a Reynolds number varying between 11 and 44, Nizkaya et al. [17] numerically found that oblates choose to log-roll regardless of their initial position and orientation.

#### 3.2.6. Applications

Works conducted during the last two decades on inertial focusing have shown that this technique has a great potential in terms of practical applications. The most developed application of inertial focusing of non-spherical particles is separation.

Particles of different shapes (hence different *λ*) but the same volume have different rotational diameters, which thus modifies the corresponding confinement ratio. As seen in Section 3.2.3, an alteration in *κ* induces a change in the particle equilibrium position. Some works took advantage of this effect to perform a so-called “shape-based” separation of biological particles. Mach and Di Carlo [58] designed an inertial microfluidic device to separate pathogenic bacteria cells from diluted blood. After two passes of the single channel system, the device removed 80% of the pathogenic bacteria and enriched red blood cells concentration four times. Two similar works were done using more sophisticated separator designs, permitting the separation of yeast cells [49] and *E*. *gracilis* [57] of different aspect ratios, with high levels of purity (more than 90%). 

Although the separation in these works is presented as a “shape-based” separation, it is still not clear if the difference in the equilibrium positions is due to a change in the aspect ratio *λ* or in the confinement degree *κ*. Thus, complementary experiments should be performed on particles with identical *κ* but different aspect ratios *λ* to verify if they have the same *Y_eq_* or not. In the case where the equilibrium positions of particles of different shapes are identical, kinetic separation based on the migration velocity could be considered, exploiting for example the observations from Nizkaya et al. [17] regarding the relation between the transitory rotational mode of the particles and their aspect ratio.

## 4. Conclusions

This review aimed to summarize and compare recent works in the literature dealing with the transport of non-spherical particles in straight square microchannels. For this purpose, a preliminary analysis of the main studies on non-spherical particles in unconfined shear flows has been conducted to identify the main parameters influencing their rotational behavior.

These different works have highlighted that in a Poiseuille flow, a particle simultaneously rotates and translates. At moderate Reynolds numbers (0 < *Re* < 150–200 depending on the configuration), near the channel entrance, the particle undergoes a transition phase. During this transition phase, it laterally migrates toward one of the equilibrium positions located near the channel walls, and its rotational mode gradually shifts from kayaking to tumbling if its aspect ratio is above 1 (prolate spheroids, rods, fibers…) or to log-rolling if its aspect ratio is below 1 (oblate spheroids, discs…). At the end of the transition period, the particle that is focused on its equilibrium position translates solely in the streamwise direction and tumbles or log-rolls with its axis of rotation parallel to the closest wall. The channel length needed for a particle to reach its equilibrium position can be different, depending on the particle aspect ratio, from that needed to reach its equilibrium rotational mode. The particles symmetry, aspect ratio, volume fraction, confinement ratio, and Reynolds number play a major role in both the rotational behavior and the translational migration of the particles. At higher Reynolds numbers, other rotational modes (steady state, inclined rolling…) and focusing positions can appear, but the different existing studies do not converge on these points. 

Although several numerical works have studied both the rotational and translational motions of non-spherical particles flowing in square channels, there is a real lack of experimental investigations in that field. To our knowledge, solely Hur et al. [55] have published experimental data on this problem. De facto, the experimental analysis of the particles’ behavior is much more complicated for non-spherical particles than for spherical ones, since a three-dimensional approach is needed to observe simultaneously the particles’ position and orientation. The recent advances in image-based 3D reconstruction techniques could be advantageously exploited here to bring new experimental insights on this subject.

All the results presented in this work are essential to better understand the particles’ transport in a Poiseuille flow and demonstrate that shape-based separation is possible. However, for that purpose, three important aspects still need to be clarified and should be the object of the coming works in that field:

- The particles’ behavior (rotational and translational) at high Reynolds numbers (over 200), which is still a subject of discussion in the literature;
- The role of the interaction between non-spherical particles on their lateral migration and longitudinal ordering, which has not been studied yet to the best of our knowledge, although it has been proven to be essential in the case of spherical particles;
- The coupling between the lateral migration and the rotation of the particle during the transitional phase, which is not yet completely understood.

Overcoming these three remaining points is essential to develop shape-based separation devices that could be integrated into Lab-on-Chip platforms.

## Figures and Tables

**Table 1:** References dealing with suspensions of non-spherical particles cited in this review paper. These works are classified according to the studied phenomenon, the type of flow, and the type of approach adopted by the investigators.

Studied Phenomenon	Rotational Behavior			Inertial Migration
Type of flow *Type of study*	Unbounded shear	Bounded shear	Poiseuille	
*Theoretical*	[15,19,20,21,22,23,24,25,26]	[27,28,29,30,31]	[22,29]	-
*Numerical*	[15,23,25,26,32,33,34,35]	[36,37,38,39,40,41,42,43,44]	[16,17,45,46,47,48]	[16,17,45,47,49]
*Experimental*	-	[27,28,29,30,31,50,51,52,53,54]	[29,48,51,55,56]	[49,55,57,58]

**Table 2:** Summary of the key conclusions concerning the rotational behavior of ellipsoidal and axisymmetric non-ellipsoidal particles. *Ka* means “kayaking”, *Tu* means “tumbling”, *LR* means “log-rolling”, *SS* means “steady state”, *DNC* means “studies do not converge”, and X means “no work identified in the literature”. The papers analyzed to produce this table are listed in the second and third columns of Table 1.

**Particle**	**Ellipsoid**		**Axisymmetric**
	Prolate (*λ* > 1)	Oblate (*λ* < 1)	Rod/cylinder (*λ* > 1)
***Re* (increasing)**	*Ka*→*Tu*→DNC	*Ka*→*LR*→DNC	*Ka*→*Tu*→*SS*
**Walls/*κ***	No effect, if the particle is far from the wall		Low *κ*: no effect High κ: effect on the transition *Re*
**Buoyancy**	Effect on the transition *Re*		X
***λ* (getting far from 1)**	Period of rotation increases		
**Initial orientation**	Effect present for *Re* = 0 DNC for *Re* > 0		X
**Concentration (increasing)**	X		The transition (*Ka*→*Tu*) occurs faster
